# Cardiomiopatia PRKAG2

**DOI:** 10.36660/abc.20220694

**Published:** 2022-11-01

**Authors:** Eduardo Back Sternick

**Affiliations:** 1 Hospital Biocor Rede D’Or São Luís Nova Lima MG Brasil Hospital Biocor, Rede D’Or São Luís, Nova Lima, MG – Brasil; 2 Hospital Governador Israel Pinheiro Belo Horizonte MG Brasil Hospital Governador Israel Pinheiro (IPSEMG), Belo Horizonte, MG – Brasil; 3 Hospital Mater Dei Belo Horizonte MG Brasil Hospital Mater Dei, Belo Horizonte, MG – Brasil

**Keywords:** Arritmias Cardíacas, Flutter Atrial, Hipertrofia Ventricular Esquerda, Cardiomiopatia Hipertrófica, Bloqueio Atrioventricular, Doença de Depósito de Glicogênio

Magalhães et al.,^1^ avaliaram as características clínicas, eletrocardiográficas e eletrofisiológicas do flutter atrial em pacientes com e sem cardiomiopatia por PRKAG2.^1^ Embora o sequenciamento genético não tenha sido realizado em seus pacientes controle, a ausência de características clínicas dessa patologia é uma evidência aceitável de um estado não portador. Em humanos, mutações no gene PRKAG2 resultam em um fenótipo altamente penetrante. É dominado pelas características cardíacas de hipertrofia ventricular esquerda, pré-excitação ventricular, taquiarritmia atrial, doença de condução cardíaca e armazenamento de glicogênio no miocárdio. Seria muito improvável encontrar um paciente com um genótipo positivo para PRKAG2 no cenário de um fenótipo negativo.

Nosso grupo foi pioneiro no estudo da cardiomiopatia PRKAG2 no Brasil. O primeiro paciente foi avaliado em 1994, um homem de 36 anos, hipertenso leve, com episódios recorrentes de flutter atrial comum. O eletrocardiograma mostrou pré-excitação ventricular, aumento das ondas P, aumento significativo da voltagem do QRS e bradicardia sinusal.^2^ De 8 irmãos, seis apresentaram achados eletrocardiográficos muito semelhantes. A mãe havia feito implante de marcapasso aos 42 anos. O ecocardiograma mostrou forma não obstrutiva de hipertrofia assimétrica do ventrículo esquerdo e discreto aumento do átrio esquerdo. Havia elementos suficientes para permitir um diagnóstico presuntivo de uma fenocópia de cardiomiopatia hipertrófica! Em 1994, porém, não conseguimos fazer o diagnóstico. As mutações do gene PRKAG2 ainda não haviam sido relatadas em 1994, mas seria 7 anos depois.^3^ Então, em 2004, realizamos o sequenciamento genético dos pacientes. Apresentamos nossa experiência durante o Congresso NASPE de 2005. Nossa pesquisa recebeu o primeiro prêmio em arritmia clínica durante o Congresso Brasileiro de Arritmias Cardíacas de 2004.^2^

Após a apresentação, alguns colegas da Bahia e de Campinas me contaram que tinham casos semelhantes. Com a ajuda do geneticista Ramon Brugada, trabalhava no Masonic Medical Research Laboratory em Nova York, obtivemos o sequenciamento genético de alguns de nossos pacientes. A mutação encontrada na maioria dos casos foi a Arg302Gln (R301Q), responsável por metade dos casos relatados na literatura (em torno de 300).^4^ Recentemente relatamos uma nova variante patogênica, H401Q.^5^ Outra nova variante, K290I, foi relatada em uma família de Bahia.^6^ Nos últimos 5 anos, o sequenciamento genético vem sendo realizado no Laboratório de Biologia Molecular do Dr. Fernando Eugenio Cruz do Instituto de Cardiologia, Rio de Janeiro. Também temos colaborado com a equipe do Prof. Campos de Carvalho da Universidade Federal do Rio de Janeiro, com o objetivo de induzir cardiomiócitos de células-tronco pluripotenciais com o propósito de entender melhor as alterações eletrofisiológicas e desenvolver técnica de edição genica.^7^

O trabalho de Magalhães et al.,^1^ contribui para uma maior visibilidade da cardiomiopatia PRKAG2. Agora acompanhamos uma coorte de 60 indivíduos de 7 famílias de diferentes lugares. Muitos desses pacientes jovens, com idades entre 20 e 30 anos, apresentam flutter atrial e foram devidamente encaminhados para ablação por cateter. Apesar de ser tratada em hospitais com alto padrão de atendimento, mesmo Hospitais Universitários, o diagnóstico da cardiomiopatia pelo PRKAG2 geralmente não é realizado. Outra característica digna de comentário é a pré-excitação ventricular, muito comum nessa síndrome. Quando presente deve ser devidamente identificada para evitar ser alvo de ablação. Uma tentativa de ablação pode resultar na produção inadvertida de bloqueio atrioventricular.^4^

É de extrema importância distinguir a cardiomiopatia por PRKAG2 da cardiomiopatia hipertrófica sarcomérica. Isso ocorre porque não apenas a apresentação clínica, mas também os resultados a longo prazo são diferentes. A morte súbita é mais prevalente na variante PRKAG2. Até mesmo o mecanismo da morte súbita é distinto. Em pacientes jovens, o flutter ou a fibrilação atrial com frequência ventricular rápida, são gatilhos para morte súbita cardíaca. Naqueles que atingem a quarta década, o bloqueio de condução atrioventricular é a principal causa, e a inserção de um marcapasso salva vidas. Frequências ventriculares rápidas durante taquiarritmias atriais estão ligadas a uma via fascículo-ventricular de condução rápida, uma variante que produz pré-excitação. Relatamos recentemente que essas vias acessórias são onipresentes em humanos.^8^ Naqueles com mutações PRKAG2, as vias provavelmente se manifestam por causa do armazenamento de glicogênio em seus cardiomiócitos.^9^

Outra característica interessante da cardiomiopatia por PRKAG2 é a ausência de fibrose ventricular.^10^ Utilizando ressonância magnética cardíaca, identificamos realce tardio por gadolínio em um sexto de nossa coorte de 30 pacientes, em comparação com uma incidência prevista em metade dos indivíduos com cardiomiopatia hipertrófica.^11^ Temos visto pacientes com mais de 50 anos, geralmente com hipertrofia significativa, que desenvolvem fibrose como mostrado no realce de gadolínio e que apresentam arritmias malignas (observações não publicadas). Em contraste, indivíduos mais velhos com a variante sarcomérica da cardiomiopatia hipertrófica parecem ter um melhor prognóstico.

A síndrome PRKAG2 deve sempre ser considerada como diagnóstico diferencial em pacientes jovens com flutter ou fibrilação atrial. Este é particularmente o caso na presença de anormalidades adicionais, como bradicardia sinusal persistente, anormalidades de condução intra-atrial ou atrioventricular, pré-excitação ventricular ou hipertrofia cardíaca inexplicável.^12,13^ Como a condição tem alta penetrância e prevalência, outros indivíduos afetados costumam ser encontrados na família. Ocasionalmente, haverá parentes com marca-passo e outros que morreram subitamente. Sendo 100% sensível, o sequenciamento genético é o padrão ouro para o diagnóstico. Se não estiver disponível, amostras de biópsia endomiocárdica percutânea do septo ventricular, avaliadas por microscopia eletrônica de transmissão, são diagnósticas na maioria dos casos. Os principais achados serão a presença de grandes depósitos de grânulos de glicogênio no citosol, a ausência de inflamação e fibrose e uma arquitetura cardiomiocítica normal.^4,14^

Para resumir, a síndrome PRKAG2 apresenta-se como uma cardiomiopatia isolada, embora algumas variantes raras tenham sido associadas a miopatias esqueléticas leves. A hipertensão arterial sistêmica é comum, e a resistência à insulina tem sido relatada, juntamente com níveis elevados de triglicerídeos. A obesidade foi relatada em modelos murinos transgênicos.^15^
