# Supplementary material for: Cardiomiopatia PRKAG2
Source: Arq Bras Cardiol. 2022 Nov 1;119(5):689–90. [Article in Portuguese] doi: 10.36660/abc.20220694 (PMC9750224; doi:10.36660/abc.20220694)
Supplement: Translation

# Translation: PRKAG2 Cardiomyopathy

**Authors:** Eduardo Back Sternick

**Affiliations:** 1 Nova Lima MG Brazil Hospital Biocor, Rede D’Or São Luís, Nova Lima, MG – Brazil; 2 Belo Horizonte MG Brazil Hospital Governador Israel Pinheiro (IPSEMG), Belo Horizonte, MG – Brazil; 3 Belo Horizonte MG Brazil Hospital Mater Dei, Belo Horizonte, MG – Brazil

**Keywords:** Arrhythmias, Cardiac, Atrial Flutter, Hypertrophy, Left Ventricular, Cardiomyopathy, Hypertrophic, Atrioventricular Block, Glycogen Storage Disease

Magalhães et al.^1^ assessed atrial flutter’s clinical, electrocardiographic, and electrophysiologic characteristics in patients with and without PRKAG2 cardiomyopathy.^1^ Although genetic sequencing was not carried out in their control patients, the absence of clinical features of this pathology is acceptable evidence of a non-carrier state. In humans, mutations in the PRKAG2 gene result in a highly penetrant phenotype. It is dominated by the cardiac features of left ventricular hypertrophy, ventricular pre-excitation, atrial tachyarrhythmia, cardiac conduction disease, and myocardial glycogen storage. It would be very unlikely to find a patient with a positive genotype for PRKAG2 in the setting of a negative phenotype.

Our group pioneered the study of PRKAG2 cardiomyopathy in Brazil. The first patient was assessed in 1994, a 36-years old male, with mild hypertension, with recurrent episodes of common atrial flutter. The electrocardiogram showed ventricular pre-excitation, enlarged P waves, a significant increase in the QRS voltage, and sinus bradycardia.^2^ Of 8 brothers, six had very similar electrocardiographic findings. Their mother had had a pacemaker implanted at the age of 42. The echocardiogram showed a non-obstructive form of asymmetric left ventricular hypertrophy and mild left atrial enlargement. There were sufficient elements to allow a presumptive diagnosis of a phenocopy of hypertrophic cardiomyopathy! In 1994, however, we were not able to make the diagnosis. The mutations of the PRKAG2 gene had yet to be reported in 1994, but it would be 7 years later.^3^ Then, in 2004, we undertook genetic sequencing of the patients. We presented our experience during the NASPE Congress of 2005. Our research received the first prize in clinical arrhythmia during the Brazilian Congress of Cardiac Arrhythmias of 2004.^2^

After the presentation, some colleagues from Bahia and Campinas told me they had similar cases. With the help of the geneticist Dr Ramon Brugada who at that time worked at Masonic Medical Research Laboratory in New York, we obtained genetic sequencing from some of our patients. The mutation found in most cases was Arg302Gln (R301Q), responsible for half of all cases reported worldwide, which now number around 300.^4^ Recently we reported a new pathogenic variant, H401Q.^5^ Another new variant, K290I, was reported in a family from Bahia.^6^ Over the last 5 years, genetic sequencing has been carried out at the Molecular Biology Laboratory of Dr. Fernando Eugenio Cruz at the Instituto de Cardiologia, Rio de Janeiro. We have also been collaborating with the team of Prof. Campos de Carvalho at the Federal University of Rio de Janeiro, aiming to induce pluripotential stem cell cardiomyocytes with the objective of better understanding the electrophysiologic changes and developing gene editing techniques.^7^

The work of Magalhães et al.^1^ serves to increase our awareness of PRKAG2 cardiomyopathy. We now follow up a cohort of 60 individuals from 7 families from different places. Many of these young patients, aged 20 to 30, have an atrial flutter and have been appropriately referred for catheter ablation. Despite being treated in hospitals with a high standard of care, even University Hospitals, the diagnosis of PRKAG2 cardiomyopathy is usually missed. Another feature worthy of comment is ventricular pre-excitation, which is very common in this syndrome. If present, this arrhythmia should properly be identified to avoid being targeted for ablation. An attempt at ablation could result in the inadvertent production of atrioventricular block.^4^

It is of utmost significance to distinguish PRKAG2 cardiomyopathy from sarcomeric hypertrophic cardiomyopathy. This is because not only the clinical presentation but also the long-term outcomes are different. Sudden death is more prevalent in the PRKAG2 variant. Even the mechanism of sudden death is distinct. In young patients, atrial flutter, or fibrillation with a fast ventricular rate, is the common trigger for sudden cardiac death. In those reaching the fourth decade, atrioventricular conduction block is the major cause, and insertion of a pacemaker is life-saving. Fast ventricular rates during atrial tachyarrhythmias are linked to a fast-conducting fasciculo-ventricular pathway, one variant that produces pre-excitation. We have recently reported that such accessory pathways are ubiquitous in humans.^8^ In those with PRKAG2 mutations, the pathways probably manifest because of the glycogen storage in their cardiomyocytes.^9^

Another interesting characteristic of PRKAG2 cardiomyopathy is the lack of ventricular fibrosis.^10^ Using cardiac magnetic resonance imaging, we have identified late gadolinium enhancement in one-sixth of our cohort of 30 patients, as compared to an anticipated incidence in half of the individuals with sarcomeric hypertrophic cardiomyopathy.^11^ We have seen patients over 50 years of age, usually with significant hypertrophy, who develop fibrosis as shown on gadolinium enhancement, and who have malignant arrhythmias (unpublished observations). In contrast, older individuals with the sarcomeric variant of hypertrophic cardiomyopathy seem to have a better prognosis.

The PRKAG2 syndrome should always be considered as a differential diagnosis in young patients with atrial flutter or fibrillation. This is particularly the case in the presence of additional abnormalities, such as persistent sinus bradycardia, intra-atrial or atrioventricular conduction abnormalities, ventricular pre-excitation, or unexplained cardiac hypertrophy.^12,13^ As the condition has a high penetrance and prevalence, other affected individuals are usually found in the family. Occasionally, there will be relatives with a pacemaker and others who died suddenly. Being 100% sensitive, genetic sequencing is the gold standard for diagnosis. If not available, percutaneous endomyocardial biopsy samples from the ventricular septum, assessed using transmission electron microscopy, are diagnostic in most cases. The key findings will be the presence of large deposits of glycogen granules in the cytosol, the absence of inflammation and fibrosis, and a normal cardiomyocytic architecture.^3,14^

To summarize, the PRKAG2 syndrome presents as an isolated cardiomyopathy, although some rare variants were associated with mild skeletal myopathies. Systemic arterial hypertension is common, and insulin resistance has been reported, along with increased levels of triglycerides. Obesity has been reported in transgenic murine models.^15^
